# A simple trick for supinated hand positioning during hand surgery

**DOI:** 10.1308/rcsann.2024.0118

**Published:** 2025-04-08

**Authors:** MA Imam, C Hutton

**Affiliations:** ^1^ Ashford and St Peter’s NHS Trust, UK; ^2^ University of East London, UK; ^3^ New Victoria Hospital, UK

## Background

Carpal tunnel syndrome is the most common peripheral nerve entrapment syndrome worldwide. It is also the entrapment neuropathy most frequently encountered by upper extremity surgeons.^1^ Carpal tunnel decompression and other surgical procedures undertaken in the palm are commonly done without an assistant. We describe a simple technique using a Trethowan Bone Lever Curved Ring Handle to keep the hand supinated, enabling the surgeon to undertake procedures on the palm without assistance.

## Technique

This simple technique would enable surgeons to operate on the palm without assistance. Even in the presence of an assistant, this simple technique will allow the assistant to help in other steps in the surgical procedure.

**Figure 1:**
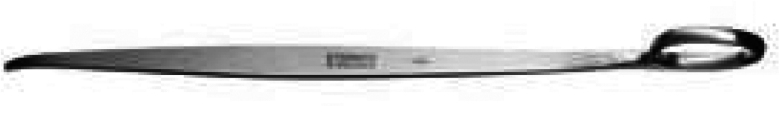
Trethowan Bone Lever Curved Ring Handle

**Figure 2:**
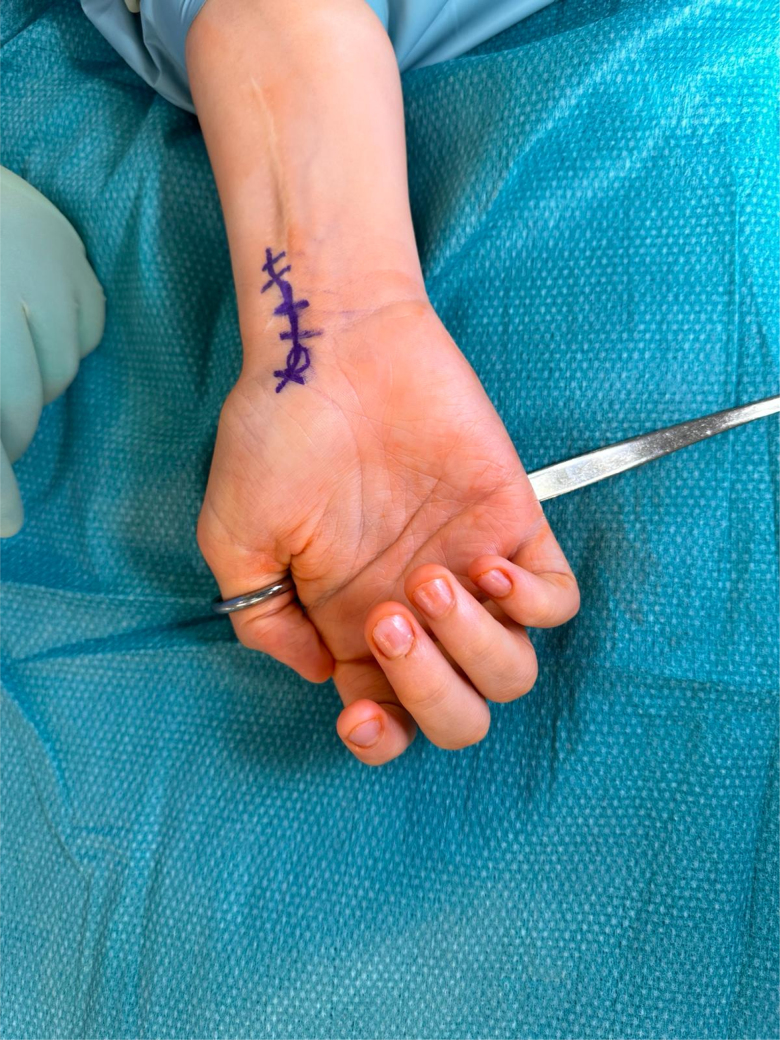
Hand kept in place supinated using the Trethowan Bone Lever Curved Ring Handle

**Figure 3:**
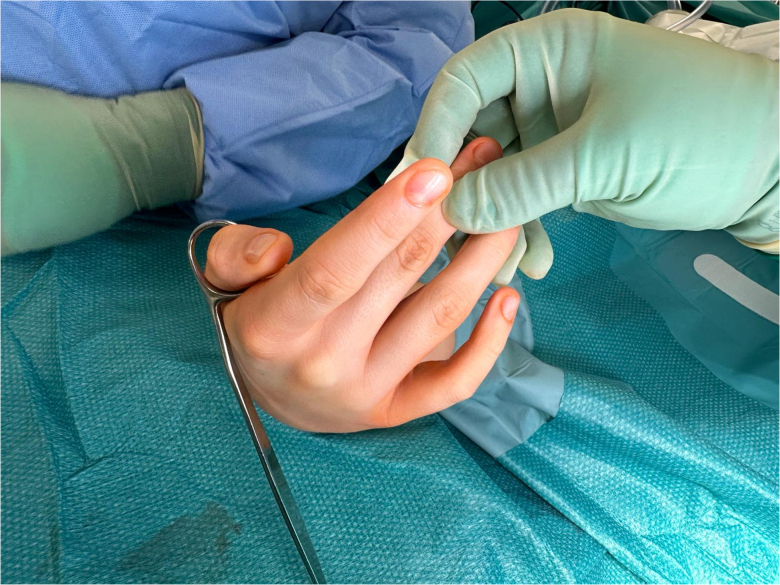
Position and orientation of the Trethowan Bone Lever Curved Ring Handle from the dorsal aspect of the hand

A Trethowan Bone Lever Curved Ring Handle (Figure 1), or ring handle spike retractor, is present in most basic surgical sets, and holds the thumb to the operation table. The thumb is held in the ring of the retractor, enabling it to stay in place and fully supinated when surgical procedures are undertaken on the palm (Figures 2 and 3). The advantages of this technique are:

- ease of access to the palm;
- comfortable surgeon position for procedures undertaken on the palm;
- this trick utilises an instrument commonly available in all basic surgical sets;
- no need for an assistant.

## Discussion

We found this setup effective at lowering operation times. This simple technique uses a Trethowan Bone Lever Curved Ring Handle. Leonardo da Vinci once said simplicity is the ultimate sophistication, and simple steps like these can simplify surgery.
